# Deep learning–based velocity antialiasing of 4D‐flow MRI

**DOI:** 10.1002/mrm.29205

**Published:** 2022-04-05

**Authors:** Haben Berhane, Michael B. Scott, Alex J. Barker, Patrick McCarthy, Ryan Avery, Brad Allen, Chris Malaisrie, Joshua D. Robinson, Cynthia K. Rigsby, Michael Markl

**Affiliations:** ^1^ Department of Biomedical Engineering Northwestern University Evanston Illinois USA; ^2^ Department of Radiology Northwestern Medicine Chicago Illinois USA; ^3^ Anschutz Medical Campus University of Colorado Aurora Colorado USA; ^4^ Division of Cardiac Surgery Northwestern Medicine Chicago Illinois USA; ^5^ Department of Medical Imaging Lurie Children's Hospital of Chicago Chicago Illinois USA

**Keywords:** 4D flow, MRI, hemodynamics, machine learning, MRI, thoracic aorta

## Abstract

**Purpose:**

To develop a convolutional neural network (CNN) for the robust and fast correction of velocity aliasing in 4D‐flow MRI.

**Methods:**

This study included 667 adult subjects with aortic 4D‐flow MRI data with existing velocity aliasing (*n* = 362) and no velocity aliasing (*n* = 305). Additionally, 10 controls received back‐to‐back 4D‐flow scans with systemically varied velocity‐encoding sensitivity (*vencs*) at 60, 100, and 175 cm/s. The no‐aliasing data sets were used to simulate velocity aliasing by reducing the venc to 40%–70% of the original, alongside a ground truth locating all aliased voxels (153 training, 152 testing). The 152 simulated and 362 existing aliasing data sets were used for testing and compared with a conventional velocity antialiasing algorithm. Dice scores were calculated to quantify CNN performance. For controls, the venc 175‐cm/s scans were used as the ground truth and compared with the CNN‐corrected venc 60 and 100 cm/s data sets

**Results:**

The CNN required 176 ± 30 s to perform compared with 162 ± 14 s for the conventional algorithm. The CNN showed excellent performance for the simulated data compared with the conventional algorithm (median range of Dice scores CNN: [0.89–0.99], conventional algorithm: [0.84–0.94], *p* < 0.001, across all simulated vencs) and detected more aliased voxels in existing velocity aliasing data sets (median detected CNN: 159 voxels [31–605], conventional algorithm: 65 [7–417], *p* < 0.001). For controls, the CNN showed Dice scores of 0.98 [0.95–0.99] and 0.96 [0.87–0.99] for venc = 60 cm/s and 100 cm/s, respectively, while flow comparisons showed moderate‐excellent agreement.

**Conclusion:**

Deep learning enabled fast and robust velocity anti‐aliasing in 4D‐flow MRI.

**Abbreviations**
CNN: convolutional neural network
AAo: ascending aorta
DAo: descending aorta
venc: velocity‐encoding sensivity

## 1 INTRODUCTION

Four‐dimensional flow MRI allows for the comprehensive assessment of aortic hemodynamics by acquiring time resolved 3‐directional blood flow velocities with full volumetric coverage of the aorta.^1^, ^2^ However, 4D‐flow MRI requires the preselection of a maximum expected velocity (or velocity sensitivity, *venc*) before scan execution, which determines the maximum velocity of the blood flow.^3^ If the venc is set too low, velocity aliasing (or phase wrapping) can occur with the flow velocity exceeding the prescribed venc, making accurate flow measurement and interpretation challenging.^4^ As the maximum expected velocity is rarely known before the scan, the venc tends to be set conservatively high in order to avoid velocity aliasing, or additional 2D phase‐contrast scout images are acquired to estimate the maximum expected velocity. However, velocity noise is proportional to the selected venc, and a high venc can consequently result in low velocity‐to‐noise ratio, and thus poor signal in low‐flow regions.^5^ This can be particularly challenging for various pathologies of the aorta, which can result in a high dynamic range of aortic flow velocities. For example, in patients with aortic valve stenosis, peak systolic flow jet velocities can be as high as 4–6 m/s (normal aortic peak velocities are on the order of 1.5 m/s), presenting challenges in selecting the most optimal venc in order to obtain accurate velocity data and avoid velocity aliasing.^1^, ^6^, ^7^, ^8^

To address these limitations, velocity‐unwrapping algorithms have been used to correct flow regions with velocity aliasing and to retrieve the true (unaliased) velocity value. However, detecting aliased regions can be challenging, particularly when large regions are affected or when velocity aliasing with multiple wraps (*n* > 1) has occurred.^9^ Many velocity‐unwrapping techniques have therefore attempted to implement an optimization approach, either through a region‐merging strategy or a graph cuts approach.^10^, ^11^ However, these methods are not robust in the presence of high noise and/or require initial (manual) seeding, to accurately identify regions for velocity unwrapping. Other attempts have focused on taking advantage of the spatial and temporal incongruities present in velocity wrapping of 2D or 3D phase contrast MRI as a strategy to correct for velocity aliasing.^12^, ^13^, ^14^, ^15^ These techniques detect velocity jumps larger than venc across the spatial and temporal domains and have been widely used to correct velocity aliasing across all velocity directions and slices in 4D‐flow MRI data.^13^, ^14^, ^15^ However, significant velocity jumps can be difficult to detect when the acquired venc is too low, resulting in severe aliasing across large regions of the data, preventing effective phase unwrapping.^14^

Recently, deep learning concepts such as convolutional neural networks (CNNs) have demonstrated their utility for the automation and acceleration of preprocessing and reconstruction of large medical imaging data.^16^ Convolutional neural networks have been shown to perform accurate segmentation of cardiovascular MRI data sets, such as 3D aortic, bi‐ventricular, and whole‐heart segmentation.^17^, ^18^, ^19^ Additionally, CNNs have been used for MRI denoising, reconstruction, and image restoration, such as artifact detection and the removal of ghosting artifacts.^20^, ^21^, ^22^ The goal of this study was to develop a CNN to automatically detect and correct velocity aliasing in 4D‐flow MRI studies of the thoracic aorta. In this study, we retrospectively leveraged a large database of 4D‐flow MRI data and used both simulated velocity aliasing (to generate labeled ground‐truth data with known location of all velocity‐aliased voxels) as well as data sets with existing real velocity aliasing. The performance of the resulting CNN‐based velocity anti‐aliasing technique was subsequently evaluated in a study with 10 prospectively enrolled healthy controls who underwent a series of 4D‐flow MRI scans at different venc levels. Our goal was to test the hypothesis that the CNN can detect and correct more velocity‐aliased image voxels compared with a conventional velocity anti‐aliasing algorithm.

## 2 METHODS

### 2.1 Study cohort

A total of 915 adult participants who underwent 4D‐flow MRI of the thoracic aorta between 2011 and 2019, including 786 patients with standard‐of‐care cardiothoracic MRI for aortic dilation and/or aortic valve disease and 129 healthy volunteers were retrospectively included in this study. This same cohort was used and published in a previous study of aortic phase‐contrast MRA segmentation.^17^ The objective was to identify patients who underwent aortic 4D‐flow MRI and contained complete data sets that were manually analyzed and postprocessed. Of the 915, 76 were excluded because the settings used in manual postprocessing (eddy current correction and noise masking thresholds) were not saved, and 172 were excluded because DICOM data were not readily available. A final total of 667 adult subjects (19–91 years, median = 51 years) were identified. The final cohort in this study includes 116 subjects who had been excluded in the prior study due to poor segmentation quality; these exams were reprocessed using the segmentation algorithm described in the prior study and manually reviewed for accuracy. Of the final 667 subjects, 568 patients underwent standard‐of‐care cardiothoracic MRI, while 99 healthy adult controls underwent research cardiothoracic MRI exams. This HIPAA‐compliant study was approved by the institutional review board. An additional cohort of 10 healthy controls were prospectively recruited. Each subject underwent three back‐to‐back 4D‐flow MRI scans at different venc levels: 175 cm/s, 100 cm/s, and 60 cm/s during a single MRI session. Patients were retrospectively enrolled with a waiver of consent, whereas controls provided written informed consent per institutional review board requirement.

### 2.2 Magnetic resonance imaging

All retrospectively enrolled subjects underwent 4D‐flow MRI with full coverage of the thoracic aorta (sagittal‐oblique 3D volume) using either 1.5T (*N* = 566; Aera, Avanto, or Espree; Siemens Healthineers, Erlangen, Germany) or 3T (*N* = 111; Skyra; Siemens Healthineers) MRI systems. The 4D‐flow MRI pulse‐sequence parameters were as follows: spatial resolution = 1.2–3.1 × 1.2–3.1 × 1.2–5.0 mm^3^, temporal resolution = 32.8–44.8 ms, FOV = 124–406 × 180–500 × 38–176 mm^3^, TE = 2.1–3.0 ms, TR = 4.1–5.4 ms, flip angle = 7°–25°, and venc = 60–500 cm/s. Data for all subjects were acquired during free breathing with respiratory navigator and electrocardiogram gating. For *N* = 536 subjects, the 4D‐flow scan was acquired after standard‐of‐care administration of contrast agent: Gadavist, Magnevist (Bayer Healthcare, Berlin, Germany), Multihance (Bracco Diagnostic, Cranbury, NJ), Dotarem (Guerbet, Raleigh, NC), or Ablavar (Lantheus Medical Imaging, Billerica, MA).

The cohort of 10 prospectively enrolled controls underwent a research MRI including three aortic 4D‐flow MRI scans with the following parameters: spatial resolution = 2.35–2.5 × 2.35–2.5 × 2.4–2.8 mm^3^, TE = 2.93–2.94 ms, TR = 3.86–3.87 ms, flip angle = 7°, and venc = 60–175 cm/s. Subject demographics and scan parameters are summarized in Table 1.

**TABLE 1:** Summary of demographics and scan parameters (age is reported as the median [interquartile range])

Total number (*N* = 667)	Age, years	51 [19–91]
	Sex	490 M/177F
	Venc range	150–500 cm/s
	Spatial resolution	1.6–2.2 × 1.6–2.2 × 2.0–5.0 mm^3^
	Temporal resolution	36.0–42.4 ms
4D‐flow MRI with velocity aliasing (*N* = 362)	Age	51 [19–84]
	Sex	287/85F
	Venc range	150–500 cm/s
	Spatial resolution	1.7–3.1 × 1.7–3.1 × 2.2–3.8 mm^3^
	Temporal resolution	32.8–41.6 ms
4D‐flow MRI without velocity aliasing (*N* = 305)	Age	51 [19–91]
	Sex	217 M/ 88F
	Venc range	150–350 cm/s
	Spatial resolution	1.7–2.8 × 1.7–2.8 × 2.2–5.0 mm^3^
	Temporal resolution	36.0–43.2 ms
Controls with multiple 4D‐flow MRI scans (*N* = 10)	Age	36 [28–70]
	Sex	9 M/1F
	Venc range	60–175 cm/s
	Spatial resolution	2.4–2.5 × 2.4–2.5 × 2.4–2.8 mm^3^
	Temporal resolution	45.28–45.36 ms

Abbreviations: F, female; M, male.

### 2.3 Standard 4D‐flow MRI preprocessing

**FIGURE 1:**
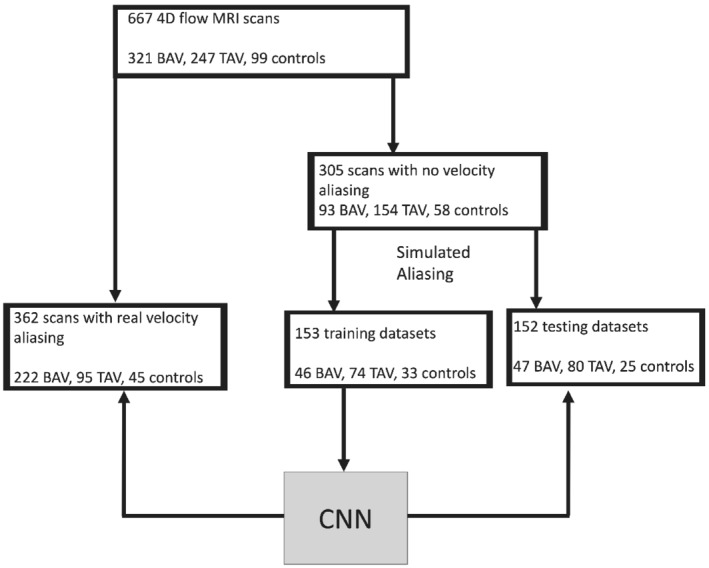
Flowchart of convolutional neural network (CNN) training and testing. The entire cohort of 667 subjects (A) was classified into either 4D‐flow MRI data with no velocity aliasing (B, *N* = 305) or data sets with real velocity aliasing present in the scan (C, *N* = 362). The data sets with no velocity aliasing were randomly divided into training (D, *N* = 153) and testing cohorts (E, *N* = 152). After CNN training, the data sets with real velocity aliasing were used for additional testing. Abbreviations: BAV, bicuspid aortic valve; TAV, tricuspid aortic valve

All 4D‐flow data underwent noise masking and corrections for phase offset errors (eddy currents, Maxwell terms) as described previously.^3^ Next, the preprocessed 4D‐flow data were used to generate 3D phase‐contrast angiogram MRA,^23^ which was used to perform manual 3D segmentation of the aorta using commercial software (Mimics, Materialize, Belgium) or automated 3D segmentation using a deep learning algorithm developed previously.^17^ All 667 4D‐flow MRI data sets (Figure 1A) were visually assessed across all slices, time frames, and velocity directions by two observers for the presence of velocity aliasing inside the aorta. Both observers needed to agree if the 4D‐flow data set contained at least one velocity‐aliased voxel in the thoracic aorta in order to be labeled as containing velocity aliasing. A total of 362 data sets (Figure 1C) were found to have at least one voxel showing velocity aliasing, whereas 305 data sets (Figure 1B) had no velocity aliasing present.

### 2.4 Ground‐truth data: simulated velocity aliasing

**FIGURE 2:**
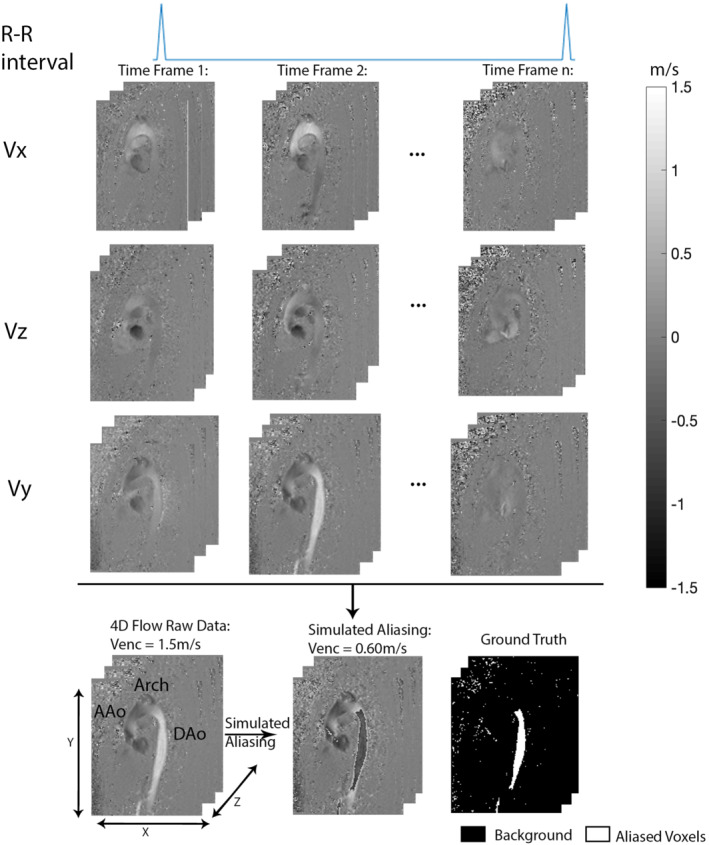
Simulated velocity aliasing for CNN training and testing. Velocity aliasing was simulated for 4D‐flow MRI data with no velocity aliasing present by reducing the venc. In this example, the original venc was 1.5 m/s, and aliasing was induced by reducing it to 0.68 m/s across all cardiac time frames, the Z‐direction, and the three velocity directions (Vx, Vy, Vz). A binary mask of the location of all aliased voxels in the 4D‐flow MRI data was used as ground truth for CNN training (bottom right: white = velocity‐aliased voxels). Abbreviations: Aao, ascending aorta; Dao, descending aorta

**FIGURE 3:**
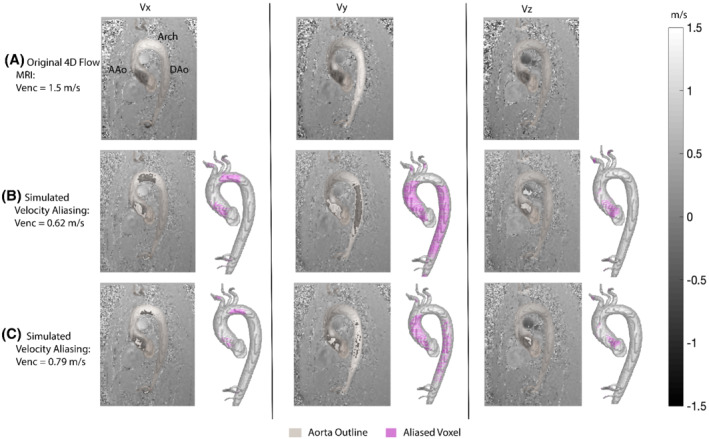
Examples of simulated velocity‐aliasing 4D‐flow MRI data. The original data set (A) was acquired with a venc of 1.5 m/s. The segmentation of the aorta (gray) was overlaid onto the data set to indicate the location of the vessel. Aliasing was simulated by lowering the venc to either 0.62 m/s (B) or 0.79 m/s (C). Each column displays a different velocity direction, and, in turn, a different pattern of velocity aliasing. To the right of each example is a 3D depiction of all aliased voxels (pink) within the aorta (gray)

As shown in Figure 1, 305 4D‐flow MRI scans with no velocity aliasing were used to generate data for CNN training and testing with known ground truth (location and number of aliased voxels). For each data set, velocity aliasing was generated by retrospectively reducing the venc to simulate aliasing. Given the known unaliased true velocity field of a 4D‐flow MRI scan, velocity aliasing was simulated as follows:

(1) $$V_{A}=V_{T}-2*{\text{venc}}_{\mathrm{sim}},\;\text{if}\;V_{T}>{\text{venc}}_{\mathrm{sim}}$$

where $V_{A}$ is the new aliased velocity; $V_{T}$ is the known velocity value of the scan; and ${\text{venc}}_{\mathrm{sim}}$ is the simulated, reduced velocity sensitivity (Figure 2). The simulated velocity aliasing was applied to the entire 4D‐flow data set across all timepoints and velocity directions. In addition, a binary mask of the location of all aliased voxels in the data set was generated, which served as the ground truth for CNN training (Figure 2, bottom right). Figure 3 provides an example of the velocity aliasing simulation. The original venc was randomly reduced twice to induce two different patterns of velocity aliasing in the data. In Figure 3B, the simulated venc (0.62 m/s) was 41% of the original venc (1.5 m/s), resulting in significant velocity aliasing throughout all three velocity directions. In Figure 3C, the simulated venc (0.79 m/s) was 53% of the original venc and resulted in less overall velocity aliasing, primarily concentrated around the ascending aorta near the aortic root. The corresponding 3D ground truth to the right in Figure 3 indicates the location of each of the aliased voxels (i.e., ground truth of number and location of aliased voxels) in pink for the aorta.

### 2.5 Convolutional neural network training and testing

The 305 4D‐flow MRI ground‐truth data (ie, simulated velocity aliasing) were randomly divided into 153 training (Figure 1D) and 152 testing data sets (Figure 1E). To systematically conduct CNN training across a wide dynamic range of velocity‐aliased data (Figure 2), the venc of each scan was randomly reduced to 40%–70% of the original venc, with each data set undergoing simulated aliasing at least four times for each epoch. For the testing data set, velocity aliasing was simulated at regulated intervals by lowering the venc to 40%, 50%, 60%, and 70% of the original venc. The 362 4D‐flow MRI data with velocity aliasing present were used as a separate testing data set.

Additionally, 10 controls with three back‐to‐back 4D‐flow MRI scans at vencs 175 cm/s, 100 cm/s, and 60 cm/s were used to compare the unwrapping performance of the CNN for the venc = 60 cm/s and 100 cm/s 4D‐flow MRI data with true (nonsimulated) in vivo ground truth (4D‐flow MRI data with no velocity aliasing for venc = 175 cm/s). Both CNN‐based velocity anti‐aliasing and the conventional algorithm were applied to 4D‐flow data with venc = 100 cm/s and 60 cm/s, and their results were compared against the 4D‐flow scan with venc = 175 cm/s.

### 2.6 Convolutional neural network architecture

The CNN used was a U‐Net network with dense blocks replacing the traditional convolutional layers as previously described.^17^ Briefly, the CNN used a series dense blocks, a collection of small convolution layers and concatenation, providing an efficient use of feature maps and CNN parameters, while retaining the encoder‐decoder design of the U‐Net network.^24^, ^25^ A composite loss function composed of a softmax cross entropy loss and a dice loss function was used for training. The CNN output was a binary mask of all detected voxels with velocity aliasing. Based on this mask, the detected wrapped voxels were then unwrapped using Eq. 1.

The CNN training was performed with a learning rate of 10^−4^, a dropout rate of 0.1, and a batch size of 1. These hyperparameters were determined using a separate validation data set in our previous work.^26^ Training occurred for 400 epochs. The input to the CNN was a 3D array of dimensions [X, Y, Z]. All inputs were centered cropped to dimensions [128, 96, Z], with the range of Z being 22–48, in order to reduce the number of noisy voxels in the data. All cardiac time frames and the three velocity directions of each 4D‐flow data set were treated as separate inputs to the CNN and compiled as a final output. The CNN was coded in *Python* 3.6.8 (Python Software, Beaverton, Oregon) using *TensorFlow* 1.12.0 (Google, Mountain View, California), and all training and testing was performed on an Intel i7‐8700K processor with a Nvidia GTX 1080‐Ti GPU. Code is provided here: https://github.com/hberhane/4D‐flow‐Velocity‐Aliasing‐CNN.

### 2.7 Conventional velocity anti‐aliasing algorithm

The velocity anti‐aliasing CNN was compared with a fully automatic conventional phase‐unwrapping algorithm.^14^ The conventional algorithm used a 3D input [X,Y,time] from the 4D‐flow data set to calculate a difference array of each voxel to nearest neighbor. This difference array was then used in determining regions with velocity jumps > venc across the spatial and temporal dimensions. The location of the velocity jump was then unwrapped using Eq. 1 to obtain the voxel's true velocity value. The algorithm was repeated for all slices and the three velocity directions, to apply anti‐aliasing across the entire 4D‐flow data set.

### 2.8 Comparative methods and statistics

To quantify CNN performance, dice scores and Hausdorff distances between the mask of detected aliased voxels from either the CNN or conventional algorithm and the ground truth for the simulated data were calculated as follows:

(2) $$\text{Dice score}=\frac{2|X\cap Y|}{\left|X\right|+|Y|}$$

(3) $$\begin{array}{cc} & \text{Hausdorff Distance}=\max\left(\max_{y\in Y}\;\left(\min_{x\in X}\left(d\left[x,y\right]\right)\right),\right.\\ & \;\left.\max_{x\in X}\;\left(\min_{y\in Y}\left(d\left[y,x\right]\right)\right)\right) \end{array}$$

where X is the binary mask of detected aliased voxels from either the CNN or conventional algorithm; Y is the ground truth; and d is the Euclidian distance. The background noise was masked out by a segmentation of the thoracic aorta, and only the phase‐wrapped voxels in the aorta were considered for dice score and Hausdorff distance calculations.

For 4D‐flow MRI data with real velocity aliasing, the numbers of aliased voxels found by the CNN or conventional method in the thoracic aorta were compared. *p*‐Values were calculated using a paired t‐test for normally distributed data or a Mann–Whitney U‐test test for nonparametric data. For all comparisons, only aliased voxels within the 3D segmentation of the thoracic aorta were counted.

Additionally, a subgroup analysis was performed to compare the performance of the CNN and the conventional algorithm in patients with varying degrees of aortic valve stenosis. The aortic stenosis was graded clinically by the peak velocity from the 2D phase‐contrast MRI (normal: <2.5 m/s; mild: 2.6–2.9 m/s; moderate: 3.0–4.0 m/s; severe: > 4.0 m/s).

For the 10 controls with multiple venc 4D‐flow MRI scans, the ground truth was determined by identifying voxels showing a difference greater than the venc (100 or 60 cm/s) between the 175‐cm/s venc (nonaliased) scan and the 100‐cm/s or 60‐cm/s venc scan. Dice scores were calculated between a mask of detected aliased voxels from the CNN or the conventional algorithm, and the ground truth in the thoracic aorta. In addition, flow quantification at the ascending aorta (Aao), aortic arch, and descending aorta (Dao) were compared between the ground truth (4D‐flow MRI with venc = 175 cm/s and no velocity) and the 4D‐flow data corrected for velocity aliasing. Three 2D analysis planes were placed at the Aao, arch and Dao, and from these planes, peak flow and net flow were obtained. Peak velocity was obtained based on regions of interest drawn at the Aao, arch, and Dao. Bland–Altman analysis was used to compared these flow and velocity metrics. Bland–Altman limits of agreement (LOA) percent difference was defined as LOA divided by the mean of the reference values of the non‐aliased, 175 cm/s venc data flow and velocity values.

Dice scores were assessed for normality using a Sharpiro‐Wilk test and were reported as either the mean ± SD if normally distributed or the median (interquartile range) if nonparametric. Likewise, an unpaired t‐test or Mann–Whitney U‐test was performed depending on the normality of the data for comparing the CNN performance with the conventional algorithm and the presence of contrast agent. Linear regression was used to assess the impact of spatial resolution on CNN performance.

## 3 RESULTS

### 3.1 Study cohort

The cohort of 667 4D‐flow MRI data sets consisted of 317 bicuspid aortic valve (BAV) patients, 251 tricuspid aortic valve (TAV) patients, and 99 healthy controls. Furthermore, our cohort contained 49 patients with severe aortic valve stenosis (AS), 17 with moderate to severe AS, 25 with moderate AS, 9 with moderate to mild AS, 24 with mild AS, and 419 patients with no AS. A total of 124 patients did not have AS grading. For the scans with no velocity aliasing present (*N* = 305), 46 BAV, 74 TAV, and 33 controls were used for CNN training, and 47 BAV, 80 TAV, and 25 controls were used for CNN testing. Finally, our cohort of data sets with real velocity aliasing consisted of 222 BAV patients, 95 TAV patients, and 45 as well as 44 patients with severe AS, 14 patients with moderate to severe AS, 20 patients with moderate AS, 6 patients with mild to moderate AS, 17 patients with mild AS, and 191 patients with no AS. For the 10 healthy controls with multiple 4D‐flow scans with different vencs, 1 subject was found to show significant movement between scans, preventing an accurate comparison between the high‐venc (175 cm/s) and low‐venc (100, 60 cm/s) scans. As such, only 9 subjects were available for analysis.

### 3.2 Convolutional neural network performance

The CNN training time was 143 ± 12 min per epoch, and the total training time was over 800 h. Application of the trained CNN for 4D‐flow velocity unaliasing resulted in processing times of 176 ± 30 s per 4D‐flow MRI data set, while the conventional algorithm took 162 ± 14 s per data set.

**FIGURE 4:**
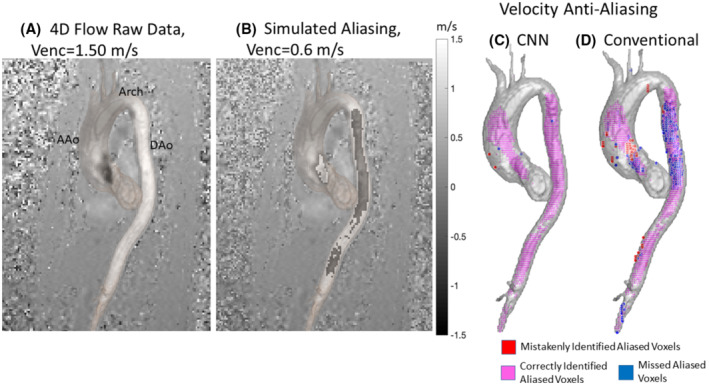
Example of a patient with simulated velocity aliasing for CNN testing. (A) On the left is the original 4D‐flow MRI scan with a venc of 1.5 m/s, showing the velocity in the head–foot (Vy) direction. (B) The 3D segmentation of the aorta (shaded surface) is overlaid on the data. Velocity aliasing was simulated by reducing the venc by 60% to 0.6 m/s. On the right is a 3D depiction of the performance of the CNN (C) and conventional algorithm (D) in detecting aliased voxels. Pink voxels indicate where the CNN or conventional algorithm correctly located velocity aliasing; blue denotes missed aliased voxels; and red shows regions where either method incorrectly identified velocity aliasing. The CNN‐based velocity anti‐aliasing was superior compared with the conventional algorithm with fewer (blue) missed and incorrectly (red) identified voxels

Figure 4 provides an example of a side‐by side comparison of a 4D‐flow data set with simulated velocity aliasing next to the original (nonaliased) ground‐truth data with venc = 1.5 m/s for an adult patient with BAV disease (Figure 4A). To simulate aliasing, the venc was reduced by 60% to 0.60 m/s (Figure 4B). A 3D representation of the detected aliased voxels within the segmented aorta for both the CNN and the conventional algorithm is shown in Figure 4C,D respectively. Correctly identified regions with velocity aliasing are shown in pink, voxels that were incorrectly identified as velocity‐aliased are shown in red, and voxels that were missed are marked in blue. Improved performance of CNN‐based velocity anti‐aliasing compared with the conventional algorithm can clearly be appreciated. Misidentified voxels from the CNN were located near the walls of the aorta, while the conventional algorithm had incorrectly identified voxels throughout the vessel.

These findings are corroborated by findings across the entire testing cohort of 152 4D‐flow MRI data sets with simulated velocity aliasing. The CNN‐based velocity anti‐aliasing outperformed the conventional algorithm as indicated in Table 2. There was a significant difference in performance between the two methods (median dice scores for CNN: 0.89 [0.62–0.97] for 0.7*venc, 0.97 [0.90–0.99] for 0.6*venc, 0.99 [0.97–0.99] for 0.5*venc, and 0.99 [0.99–0.99] for 0.4*venc; for conventional algorithm: 0.84 [0.48–0.97] for 0.7*venc, 0.93 [0.78–0.98] for 0.6*venc, 0.94 [0.85–0.98] for 0.5*venc, and 0.90 [0.79–0.95] for 0.4*venc; *p* < 0.001 across all vencs). Additionally, CNN performance improved as the number of aliased voxels in the data set increased (as the simulated venc was reduced). The CNN and conventional algorithm both performed worst on 4D‐flow MRI data with the fewest aliased voxels (70% of the original venc), where the CNN had a median Dice score of 0.89 [0.62–0.97] and the conventional algorithm had a median Dice score of 0.84 [0.48–0.97]. Additionally, we found no significant difference in CNN or conventional algorithm performance and the administration of contrast agent. Furthermore, the CNN or conventional algorithm performance was not found to be sensitive to voxel size.

**TABLE 2:** Summary of Dice scores and Hausdorff distance for the CNN and conventional algorithm across all testing data sets with simulated velocity aliasing (*N* = 152)

	Number of aliased voxels	CNN Dice score	Hausdorff distance CNN (mm)	Conventional algorithm Dice score	Hausdorff distance conventional algorithm (mm)	*p*‐Value
${\text{venc}}_{\mathrm{sim}}=0.4*\text{venc}$	11 710 [5550–22 131]	0.99 [0.99–0.99]	2.13 [0.93–4.75]	0.90 [0.79–0.95]	9.08 [3.60–13.64]	<0.001
${\text{venc}}_{\mathrm{sim}}=0.5*\text{venc}$	3519 [1302–6610]	0.99 [0.97–0.99]	2.15 [0.80–4.04]	0.94 [0.85–0.98]	7.5 [2.13–14.0]	<0.001
${\text{venc}}_{\mathrm{sim}}=0.6*\text{venc}$	725 [245–2152]	0.97 [0.90–0.99]	3.56 [1.06–10.7]	0.93 [0.78–0.98]	7.42 [3.01–20.5]	<0.001
${\text{venc}}_{\mathrm{sim}}=0.7*\text{venc}$	194 [69–615]	0.89 [0.62–0.97]	8.60 [4.75–29.9]	0.84 [0.48–0.97]	13.5 [4.93–45.6]	<0.001

*Note*: The median number of aliased voxels, and the Dice scores of the CNN and conventional algorithm are provided. The CNN showed significantly improved Dice scores and Hausdorff Distance compared with the conventional algorithm. All values are reported as the median [interquartile range].

Abbreviation: ${\text{venc}}_{\mathrm{sim}},$ simulated venc.

### 3.3 Convolutional neural network application to 4D‐flow MRI data with existing velocity aliasing

**FIGURE 5:**
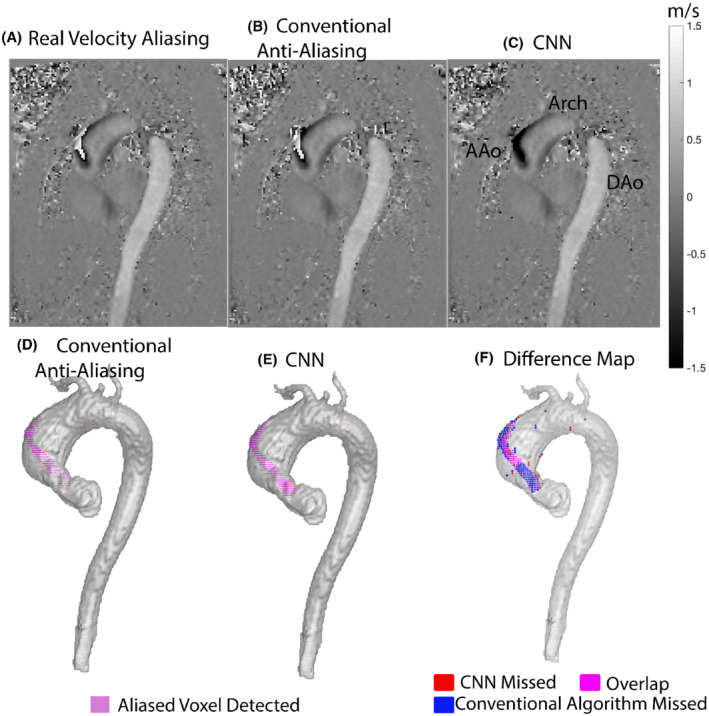
Example of a patient with real velocity aliasing for CNN testing. (A) The original velocity image is shown at left at peak systole and in the Vy direction (A). (B) The same slice after undergoing velocity anti‐aliasing with the conventional algorithm. (C) The slice after undergoing velocity anti‐aliasing using the CNN. On the bottom row are 3D visualizations of the aorta with all aliased voxels detected by each method in the Vy direction and magnified regions of the ascending aorta (pink, conventional algorithm [D] and CNN [E]). The 2D slices show that the CNN was better able to correct the aliased voxels at the edge of ascending aorta better than the conventional algorithm. (F) The 3D images provide a difference map highlighting the region in which both methods detected aliased voxels (pink), where the CNN did not detect aliased voxels but the conventional algorithm did (red), and where the CNN detected aliased voxels but the conventional algorithm did not (blue). As seen in the figure, the conventional algorithm failed to detect many voxels in the aortic root region, which the CNN was able to

**FIGURE 6:**
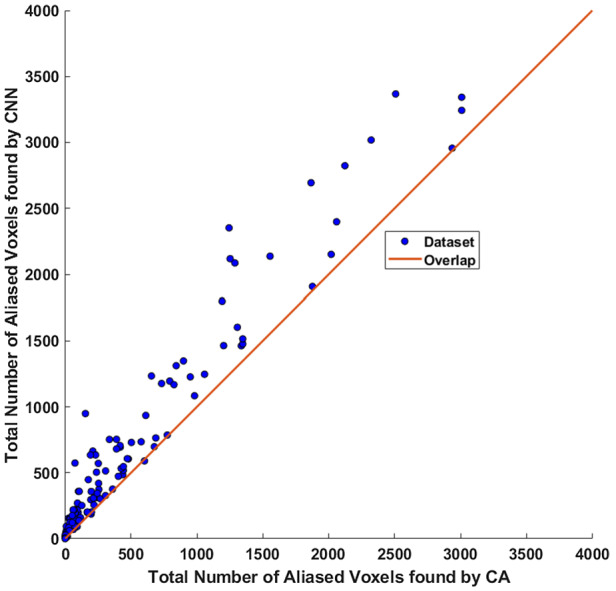
Comparison of velocity anti‐aliasing performance between CNN and conventional algorithm across all data sets with real velocity aliasing present (*N* = 362). Each 4D‐flow MRI data set is represented as a blue data point. The orange line represents identical performance of both methods (ie, same number of aliased voxels identified by both CNN and the conventional algorithm). The CNN based velocity anti‐aliasing detected as many or more aliased voxels than the conventional algorithm in 360 of 362 4D‐flow MRI data sets. Abbreviation: CA, contrast agent

Figure 5 provides an example of a 4D‐flow MRI data set with real velocity aliasing (Figure 5A; head–foot [Vy] velocity direction at peak systole) and the correction provided by the conventional algorithm (Figure 5B,D) and the CNN (Figure 5C,E). The CNN‐based velocity anti‐aliasing identified more aliased voxels at the Aao and near the edge of the vessel compared with the conventional algorithm (CNN: 3335 voxels vs conventional: 2394). These observations were confirmed across all 362 data sets with existing velocity aliasing in the original 4D‐flow MRI scan. The median number of detected aliased voxels for the CNN was 159 voxels [31–605], whereas the conventional algorithm detected a median of 65 [7–417] aliased voxels (*p* < 0.001). A comparison of the conventional algorithm and CNN is shown in Figure 6. Here, each blue dot represents a data set and is located along the axis of how many aliased voxels were detected by the CNN or the conventional algorithm. The orange line represents the same number of voxels detected by both methods (slope of 1). The CNN‐based velocity anti‐aliasing detected at least as many or more aliased voxels as the conventional algorithm in 360 of 362 4D‐flow MRI data sets.

Additionally, data sets with real velocity aliasing of patients with severe, moderate–severe, moderate, moderate–mild, and mild AS were used to further explore the CNN performance, and the results are summarized in Supporting Information Table S1. Across all AS patient groups, we found that the CNN continued to detect more aliased voxels than the conventional algorithm, showing a significant difference in performance (*p* < 0.001). The largest disparities between the two methods were found for 4D‐flow MRI in patients with moderate–mild or higher severity of AS, with the CNN detecting > 150% more aliased voxels than the conventional algorithm (*p* < 0.001). For patients with mild or no AS, the CNN was able to detect 130% and 100%, respectively, more voxels than the conventional algorithm (*p* < 0.001 for both).

### 3.4 Convolutional neural network application in healthy controls with 4D‐flow MRI data with multiple vencs

**FIGURE 7:**
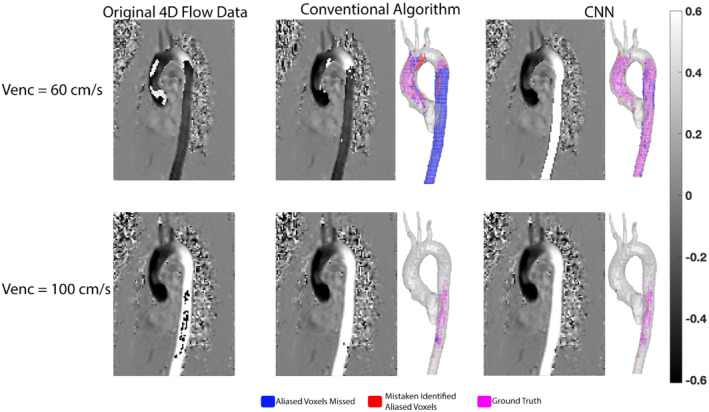
Example of real velocity anti‐aliasing comparison with CNN and conventional algorithm with known ground truth. The top row shows the results for a venc of 60 cm/s, while the bottom row is from a venc of 100 cm/s. As seen in the difference map for 60‐cm/s venc, the conventional algorithm failed to correctly identify velocity‐aliased voxels in the descending aorta, whereas the CNN was successful. However, the CNN failed to correct aliased voxels at the edge of vessel, which may be due to the partial volume effect. The Dice score for the CNN in this data set was 0.98 for venc of 60 cm/s and 0.99 for a venc of 100 cm/s, and for the conventional algorithm it was 0.75 for venc of 60 cm/s and 0.98 for venc of 100 cm/s

Figure 7 provides an example of the velocity‐aliasing correction from the CNN and the conventional algorithm for 4D‐flow data set at a venc of 100 cm/s, showing mild velocity aliasing, and at a venc of 60 cm/s, showing severe velocity aliasing. The CNN showed Dice scores of 0.98 and 0.99 for 60 cm/s and 100 cm/s venc data sets, respectively. Additionally, the conventional algorithm showed Dice scores of 0.75 and 0.98 for the 60‐cm/s and 100‐cm/s venc data sets, failing to correct aliasing in the Dao of the 60‐cm/s venc scan. Across all nine control data sets, CNN‐based velocity antialiasing showed excellent performance with a median Dice score of 0.96 [0.87–0.99] and median Hausdorff distance of 2.93 [1.04–8.5] mm at venc = 100 cm/s and 0.98 [0.95–0.99] and 2.39 [1.43–3.83] mm at venc = 60 cm/s. The conventional algorithm performed equally well for correcting mild velocity aliasing at venc = 100 (median Dice score of 0.97 [0.88–0.98] and median Hausdorff distance of 2.51 [1.31–6.81] mm), but performance was impaired for venc = 60 cm/s (Dice score of 0.76 [0.62–0.98], 11.0 [5.10–20.9] mm). For comparison, the median number of aliased voxels in the 60‐cm/s venc data was 18 950 [11 405–22 185], whereas the median number of detected aliased voxels for the CNN and the conventional algorithm were 18 544 [11 348–19 707] and 10 227[6234–12 589], respectively. For the 100‐cm/s data, the median number of aliased voxels was 787 [81–1740], whereas CNN detected 770[73–1710] and conventional algorithm detected 771[75–1680].

The results of Bland–Altman analysis for flow quantification are summarized in Table 3. For peak velocity comparisons between the 60‐cm/s venc data sets to the ground‐truth 175‐cm/s data sets, the CNN showed a low bias (−0.01 to −0.07 m/s) and LOA between 11.2% and 12.7% mean difference, whereas the conventional algorithm showed a bias between −0.03 and −0.1 m/s and LOA between 11.9% and17.1% mean difference. For net flow comparisons in the same data sets, the CNN showed a bias between 0.7 ml and1.5 ml and LOA between 10.6% and 11.8% mean difference, whereas the conventional algorithm showed a bias between −15.1 ml and 2.7 ml and LOA between 9.9% and 69.9% mean difference. For peak flow comparisons, the CNN showed a bias between −2.6 ml/s and 4.3 ml/s and LOA between 8.1% and12.2% mean difference, whereas the conventional algorithm showed a bias between −66.0 ml/s and 1.7 ml/s and LOA between 10.1% and 60.3% mean difference. Bland–Altman plots are provided for the CNN and conventional algorithm in Supporting Information Figure S1.

**TABLE 3:** Summary of the CNN and conventional algorithm hemodynamic comparisons with the ground truth across all (*N* = 9) controls

	Aao				
Flow quantifications: Mean ± SD	CNN (venc 60 cm/s)	Conventional algorithm (venc 60 cm/s)	CNN (venc 100 cm/s)	Conventional algorithm (venc 100 cm/s)	Ground truth (venc 175 cm/s)
Peak velocity (m/s)	1.23 ± 0.21	1.22 ± 0.26	1.26 ± 0.22	1.26 ± 0.21	1.30 ± 0.24
Net flow (ml)	104.4 ± 16.9	90.5 ± 22.6	101.8 ± 17.7	101.7 ± 17.5	103.8 ± 19.6
Peak flow (ml/s)	402.3 ± 91.6	331.7 ± 99.2	402.4 ± 92.9	402.7 ± 93.3	398.0 ± 86.2
Bland–Altman: bias (LOA)					
Peak velocity (m/s)	0.07 (12.7%)	0.08 (14.1%)	0.04 (10.6%)	0.03 (10.5%)	
Net flow (ml)	0.7 (10.6%)	13 (23.7%)	2.0 (10.1%)	2.2 (11.2%)	
Peak flow (ml/s)	4.3 (8.5%)	66.0 (39.1%)	4.4 (9.2%)	4.6 (9.52%)	
Flow quantifications: Mean ± SD	Arch				
Peak velocity (m/s)	1.04 ± 0.22	1.01 ± 0.19	1.1 ± 0.25	1.05 ± 0.22	1.05 ± 0.22
Net flow (ml)	73.5 ± 13.5	74.7 ± 14.8	72.6 ± 14.9	73.3 ± 16.3	72.0 ± 15.8
Peak flow (ml/s)	251.2 ± 66.2	256.3 ± 68.1	257.6 ± 64.6	260.4 ± 69.4	254.6 ± 64.6
Bland–Altman: bias (LOA)					
Peak velocity (m/s)	0.01 (11.2%)	0.03 (11.9%)	0.05 (10.9%)	0.01 (11.4%)	
Net flow (ml)	1.5 (10.7%)	2.7 (9.9%)	0.61 (11.5%)	1.3 (11.1%)	
Peak flow (ml/s)	2.6 (8.07%)	1.7 (10.1%)	3.0 (10.7%)	5.8 (10.6%)	
Flow quantifications: Mean ± SD	Dao				
Peak velocity (m/s)	1.1 ± 0.17	1.06 ± 0.12	1.15 ± 0.23	1.14 ± 0.23	1.16 ± 0.29
Net flow (ml)	78.9 ± 15.0	62.7 ± 35.7	76.3 ± 13.7	76.1 ± 13.7	77.8 ± 13.0
Peak flow (ml/s)	262.3 ± 73.3	201.7 ± 86.5	267.0 ± 64.3	263.4 ± 59.4	261.6 ± 61.7
Bland–Altman: bias (LOA)					
Peak velocity (m/s)	0.06 (11.2%)	0.1 (17.1%)	0.02 (11.6%)	0.03 (12.0%)	
Net flow (ml)	1.2 (11.8%)	15.1 (69.9%)	1.5 (8.43%)	1.8 (7.89%)	
Peak flow (ml/s)	0.65 (12.1%)	60.0 (60.3%)	5.3 (7.16%)	1.9 (5.24%)	

*Note*: For peak velocity, regions of interest were drawn at the ascending, arch, and descending aorta, while three planes at the same regions were used for the net and peak flow quantifications. Bland–Altman results are provided with the bias and the limits of agreement as the percent difference from the reference mean. The ground truth used was the venc of 175 cm/s 4D‐flow MRI data sets.

Abbreviations: Aao, ascending aorta; LOA, limits of agreement.

For the 100‐cm/s venc data sets, the CNN and the conventional algorithm showed similar performances in flow quantification comparisons with the 175‐cm/s venc data sets (Table 3, Supporting Information Figure S2). For peak velocity, the CNN had a bias between −0.04 m/s and 0.05 m/s and LOA between 10.6% and11.6% mean difference, whereas the conventional algorithm showed bias of −0.03 m/s to 0.01 m/s and LOA between 10.5% and 12.0% mean difference. For net flow, the CNN had a bias of −2.0–0.61 ml and LOA between 8.4% and 11.5% mean difference, and the conventional algorithm showed similar performance with a bias of −2.2–1.3 ml and LOA between 7.9% and 11.2% mean difference. And for peak flow, the CNN had a bias between 3.0 ml/s and 5.3 ml/s and LOA between 7.2% and 10.7% mean difference; likewise, the conventional algorithm showed a bias of 1.9–5.8 ml/s and LOA between 5.2% and 10.6% mean difference. Additionally, we assessed the time‐resolved performance of CNN and conventional algorithm across the cardiac cycle for the 60‐cm/s and 100‐cm/s venc data (Supporting Information Figures S3 and S4). Generally, the CNN performed well for both data sets especially at systole (Dice scores > 0.9); however, performance was reduced during diastole (Dice scores < 0.7) due to the limited number of aliased voxels present Supporting Information (Figures S3 and S4). For the conventional algorithm, it showed strong performance in systole for the 100‐cm/s venc data (Dice scores > 0.9), but failed to detect any voxel in diastole (Supporting Information Figure S4), while showing moderate‐to‐poor performance in the 60‐cm/s venc data across the cardiac cycle (Supporting Information Figure S3).

## 4 DISCUSSION

Our study found that our CNN performed velocity anti‐aliasing in comparable speed to a conventional anti‐aliasing algorithm; CNN‐based velocity anti‐aliasing showed excellent performance on 4D‐flow MRI data sets with simulated velocity aliasing compared with a conventional algorithm; the CNN was able to detect more aliased voxels than the conventional algorithm in data sets with real velocity aliasing. When analyzing patients with different AS status, the CNN was able to detect and correct significantly more aliased voxels across all patients, and the CNN showed excellent performance in correcting velocity anti‐aliasing in a cohort of subjects with 4D‐flow MRI scans with multiple vencs.

In the testing data set with simulated velocity aliasing, we found that the results for the highest simulated venc (0.7*venc) showed the lowest Dice scores compared with the other simulated vencs. This is likely due to the very few aliased voxels present in the data set at this simulated venc. As such, if a few aliased voxels were missed or if a few false positives were detected, there would be a significant impact on the Dice score. Clinically, the preselected venc tends to be about 10%–20% higher than the expected peak velocity in the data, and at a simulated venc of 0.7*venc, it may still be close to the peak velocity in the data.

Data in 10 prospectively enrolled healthy control subjects with back‐to‐back 4D‐flow MRI scan at three different vencs (60 cm/s, 100 cm/s, and 175 cm/s) confirmed the robust performance of our velocity‐antialiasing CNN. Scans obtained at 175 cm/s, which had no velocity aliasing, were used as the ground truth to test the performance of the CNN in detecting and correcting the velocity aliasing of the data sets with real severe velocity aliasing (venc of 60 cm/s) and low to moderate aliasing (venc of 100 cm/s). Subsequent quantification of peak velocity, net flow, and peak flow comparisons between the CNN velocity aliasing–corrected data with the ground truth (venc of 175 cm/s) demonstrated moderate to excellent agreement.

Prior attempts at velocity aliasing correction in 4D‐flow MRI have generally focused on attempting to apply techniques initially developed for 2D phase‐contrast MRI methods.^12^, ^13^, ^14^ However, our CNN was able to perform equally or better in feature extracting for the detection of aliased voxels. Alternatively, the Laplacian method was designed for 4D‐flow MRI using Laplacian operators to unwrap all three spatial directions and temporal domains.^27^ However, the algorithm fails when large regions of the data are aliased, preventing the true phase gradient from being detected.^27^ For heavily aliased data sets, our CNN demonstrated its capabilities in accurately correcting them; however, it was not directly compared against the Laplacian method.

Furthermore, our anti‐aliasing CNN was able to be effectively incorporated into a standard clinical workflow for 4D‐flow postprocessing. The CNN is fully automated, does not require any user inputs, and can be implemented directly on the scanner or a clinical workstation. Additionally, our anti‐aliasing CNN can easily be incorporated with our prior work using a CNN for aorta segmentation, providing an efficient automated 4D‐flow processing pipeline.

There are a number of limitations of our study. Our CNN was not implemented to correct velocity aliasing that occurs due to multiple phase wraps. The CNN was trained using data sets with simulated aliasing, where the aliasing was simulated with one phase wrap. As such, the CNN, in its current implementation, has difficulty in detecting voxels wrapped two or more times. In the future, we hope to implement a multilabeled CNN with training data consisting of data sets with multiwrapped aliasing. This would provide a mapping of all aliased voxels as well as a value indicating how many phase wraps to correct it. Similarly, data sets with real velocity aliasing lack a proper ground truth to properly quantify the performance of the anti‐aliasing methods. Instead, a conventional velocity anti‐aliasing technique was used as a basis of comparison as a surrogate for accuracy. Furthermore, although we did not see an impact on the CNN performance as a result of the different velocity‐to‐noise ratio between the 100‐cm/s and 60‐cm/s venc scans, the CNN was found to have difficulty in unwrapping aliased voxels on the vessel wall (Figure 7). This is likely due to the partial volume effect at the vessel wall, resulting in the voxels not experiencing full wraps (by 2π) but instead partial wrapping (<2π). Additionally, the 4D‐flow sequence used in this study is based on 4‐point encoding, consisting of a reference scan and three independent velocity sensitive scans (Vx, Vy, Vz). As such, the velocity aliasing present in the data are independent for each velocity direction, and consequently, the CNN was trained based on that assumption. Another limitation of this study is that the CNN was only compared against one velocity anti‐aliasing algorithm, and, in the future, we hope to assess the CNN's performance against various other algorithms.

## 5 CONCLUSIONS

A CNN was developed for velocity anti‐aliasing in 4D‐flow MRI, demonstrating excellent performance on data sets with simulated low venc aliasing and the ability to detect and correct more aliased voxels compared with a conventional algorithm. Future studies should extend its application to other vasculature beds, multiple centers, and MRI vendors.

## Supporting information


**Figure S1:** Bland–Altman plots for peak velocity (A), net flow (B), and peak flow (C) for the convolutional neural network CNN (top row) and conventional algorithm (bottom row) performance on the 60‐cm/s venc data set compared with the ground truth of 175 cm/s venc. Values for peak velocity are obtained from manual regions of interest (ROIs), while net and peak flow are from manually placed planes. Red dots indicate the ascending aorta (AAo); blue dots indicate the arch; and green dots indicate the descending aorta (DAo). Bland–Altman bias and limits of agreement are for all of the measurements together
**Figure S2**: Bland–Altman plots for peak velocity (A), net flow (B), and peak flow (C) for the CNN (top row) and conventional algorithm (bottom row) performance on the 100‐cm/s venc data set compared with the ground truth of 175 cm/s venc. Values for peak velocity are obtained from manual ROIs, while net and peak flow are from manually placed planes. Red dots indicate the ascending aorta (AAo); blue dots indicate the arch; and green dots indicate the descending aorta (Dao). Bland–Altman bias and limits of agreement are for all of the measurements together
**Figure S3**: The time distribution of Dice scores and the number of aliased voxels in the ground truth for the 60‐cm/s venc data. During systole (time frames 3–11), the CNN performed well (median Dice score > 0.9), although showed a decline in performance in diastole (time frames > 11; Figure S5A). This is likely due to the small number of aliased voxels present in the data during diastole (Figure S5B), which could result in a huge impact on the Dice score as a result of missing a few voxels. The conventional algorithm showed moderate to poor performance across the cardiac cycle. The dots show the median Dice score, and the bars indicate the interquartile range. For instances in which the ground truth was empty (no velocity aliasing), we calculated the Dice scores by adding a small constant (1e‐5) at both the numerator and denominator, to avoid dividing by zero
**Figure S4**: The time distribution of Dice scores and the number of aliased voxels in the ground truth for the 100‐cm/s venc data. During systole (time frames 3–11), the CNN performed well (median Dice score > 0.9), although showed a decline in performance in diastole (time frames > 11; Figure S5A). This is likely due to the small number of aliased voxels present in the data during diastole (Figure S5B), which could result in a huge impact on the Dice score as a result of missing a few voxels. The conventional algorithm, likewise, showed performance during systome but failed to detect any aliased voxels during. The dots show the median Dice score, and the bars indicate the interquartile range. For instances in which the ground truth was empty (no velocity aliasing), we calculated the Dice scores by adding a small constant (1e‐5) at both the numerator and denominator, to avoid dividing by zero
**Table S1**: Summary of the CNN and conventional algorithm comparison for stenosis patients with real velocity aliasing. Note: The patients were divided into groups based on the stenotic grading. The median number of detected velocity‐aliased voxels and the interquartile range are provided. There was a significant difference between the two methods across all patient groups, with the CNN consistently detecting more aliased voxels than the conventional algorithm.

## Data Availability

Code for the CNN are available on Github here: https://github.com/hberhane/4D‐flow‐Velocity‐Aliasing‐CNN.
