# Supplementary material for: Deep learning–based velocity antialiasing of 4D‐flow MRI
Source: Magn Reson Med. 2022 Apr 5;88(1):449–63. doi: 10.1002/mrm.29205 (PMC9050855; doi:10.1002/mrm.29205)
Supplement: Supplementary file 1 — Figure S1: Bland–Altman plots for peak velocity (A), net flow (B), and peak flow (C) for the convolutional neural network CNN (top row) and conventional algorithm (bottom row) performance on the 60‐cm/s venc data set compared with the ground truth of 175 cm/s venc. Values for peak velocity are obtained from manual regions of interest (ROIs), while net and peak flow are from manually placed planes. Red dots indicate the ascending aorta (AAo); blue dots indicate the arch; and green dots indicate the descending aorta (DAo). Bland–Altman bias and limits of agreement are for all of the measurements together Figure S2: Bland–Altman plots for peak velocity (A), net flow (B), and peak flow (C) for the CNN (top row) and conventional algorithm (bottom row) performance on the 100‐cm/s venc data set compared with the ground truth of 175 cm/s venc. Values for peak velocity are obtained from manual ROIs, while net and peak flow are from manually placed planes. Red dots indicate the ascending aorta (AAo); blue dots indicate the arch; and green dots indicate the descending aorta (Dao). Bland–Altman bias and limits of agreement are for all of the measurements together Figure S3: The time distribution of Dice scores and the number of aliased voxels in the ground truth for the 60‐cm/s venc data. During systole (time frames 3–11), the CNN performed well (median Dice score > 0.9), although showed a decline in performance in diastole (time frames > 11; Figure S5A). This is likely due to the small number of aliased voxels present in the data during diastole (Figure S5B), which could result in a huge impact on the Dice score as a result of missing a few voxels. The conventional algorithm showed moderate to poor performance across the cardiac cycle. The dots show the median Dice score, and the bars indicate the interquartile range. For instances in which the ground truth was empty (no velocity aliasing), we calculated the Dice scores by adding a small constant (1e‐5) at both the numerator a [file MRM-88-449-s001.docx]

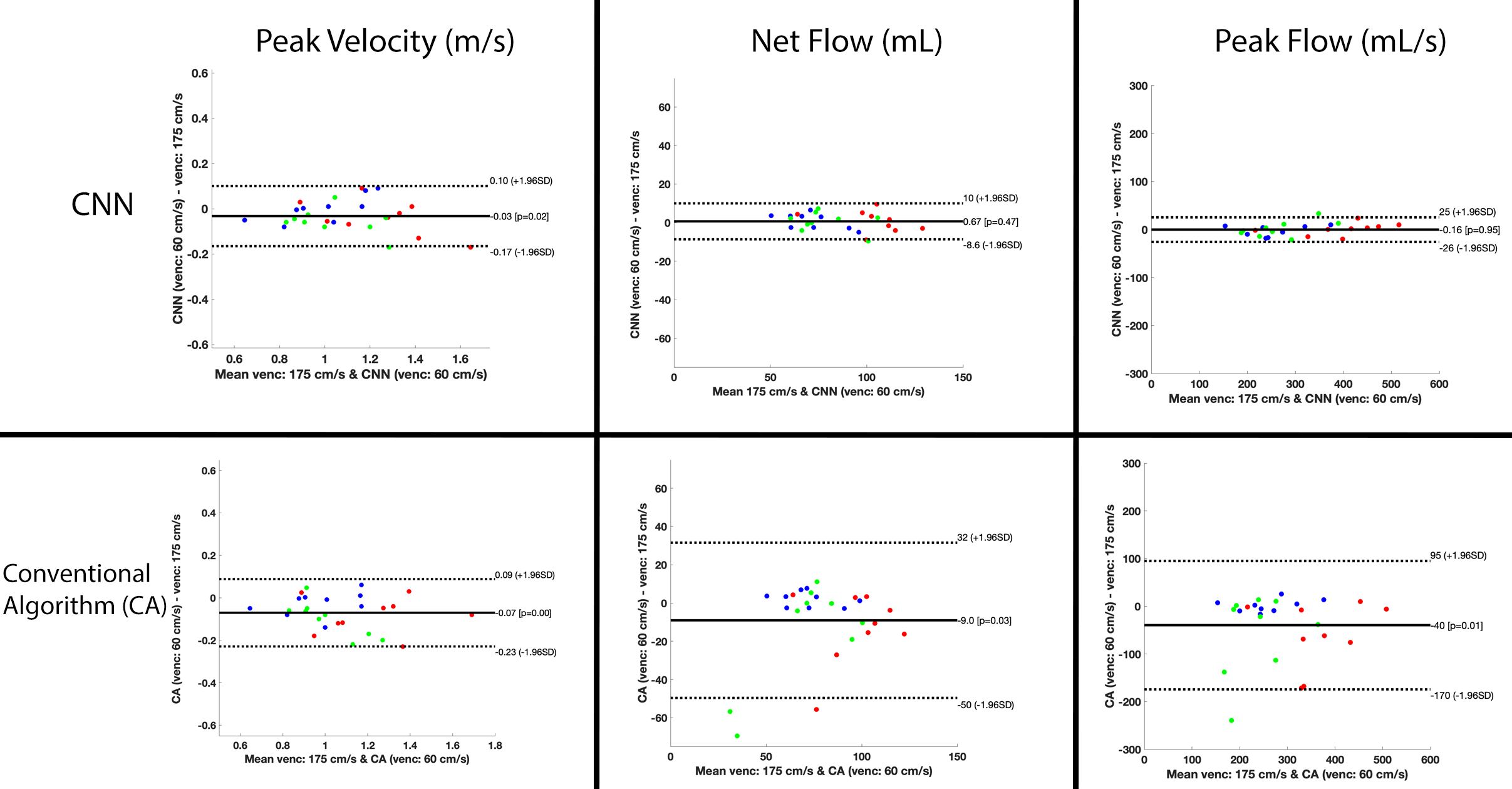


**Figure S1**: Bland-Altman plots for peak velocity (A), net flow (B), and peak flow (C) for the CNN (top row) and conventional algorithm (bottom row) performance on the 60 cm/s venc dataset compared to the ground-truth of 175 cm/s venc. Values for peak velocity are obtained from manual ROIs while net and peak flow are from manually placed planes. Red dots indicate the ascending aorta (AAo), blue dots indicate the arch, and green dots indicate the descending aorta (DAo). Bland-Altman bias and limits of agreement are for the all measurements together.


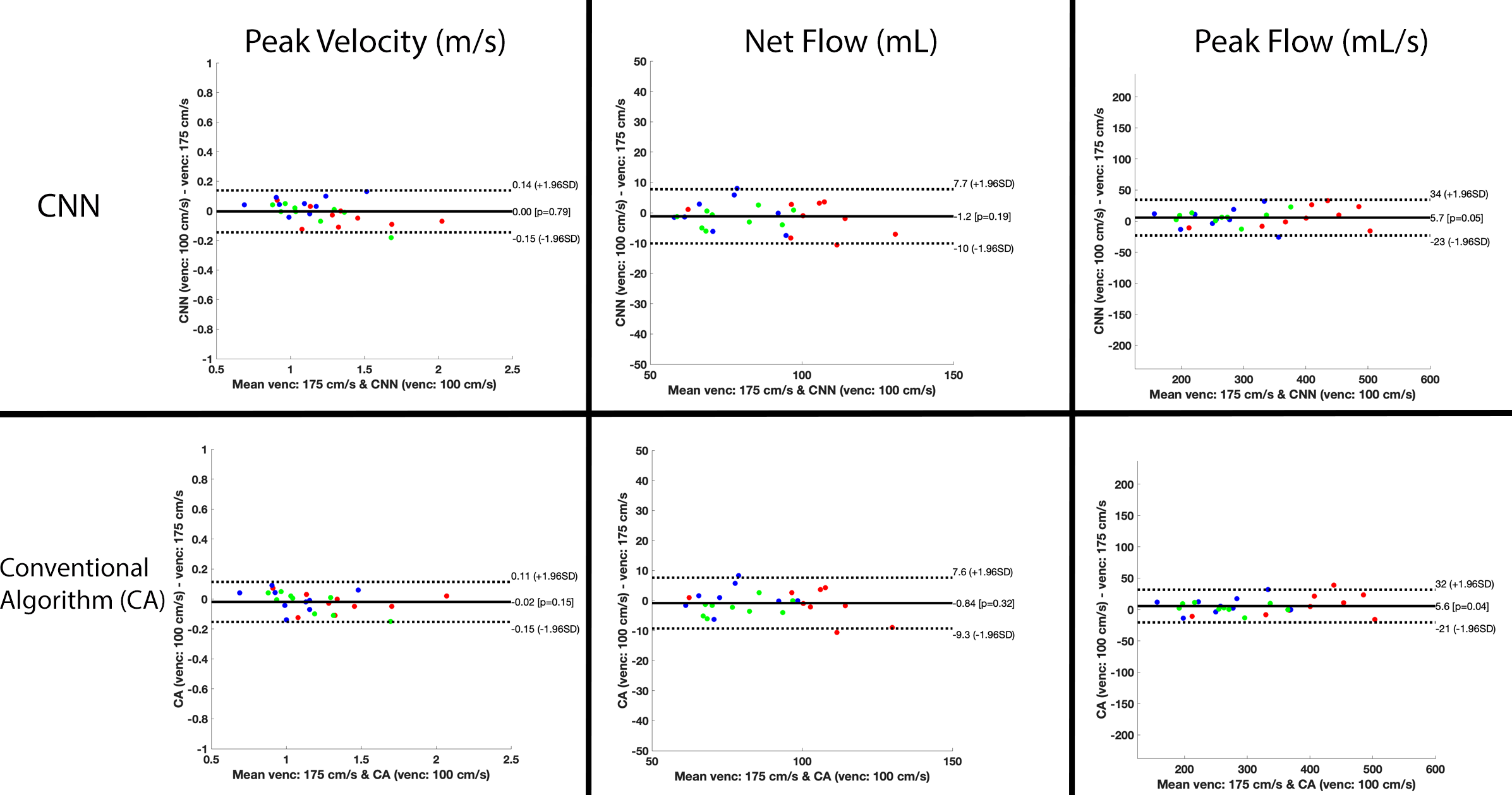


**Figure S2**: Bland-Altman plots for peak velocity (A), net flow (B), and peak flow (C) for the CNN (top row) and conventional algorithm (bottom row) performance on the 100 cm/s venc dataset compared to the ground-truth of 175 cm/s venc. Values for peak velocity are obtained from manual ROIs while net and peak flow are from manually placed planes. Red dots indicate the ascending aorta (AAo), blue dots indicate the arch, and green dots indicate the descending aorta (DAo). Bland-Altman bias and limits of agreement are for the all measurements together.


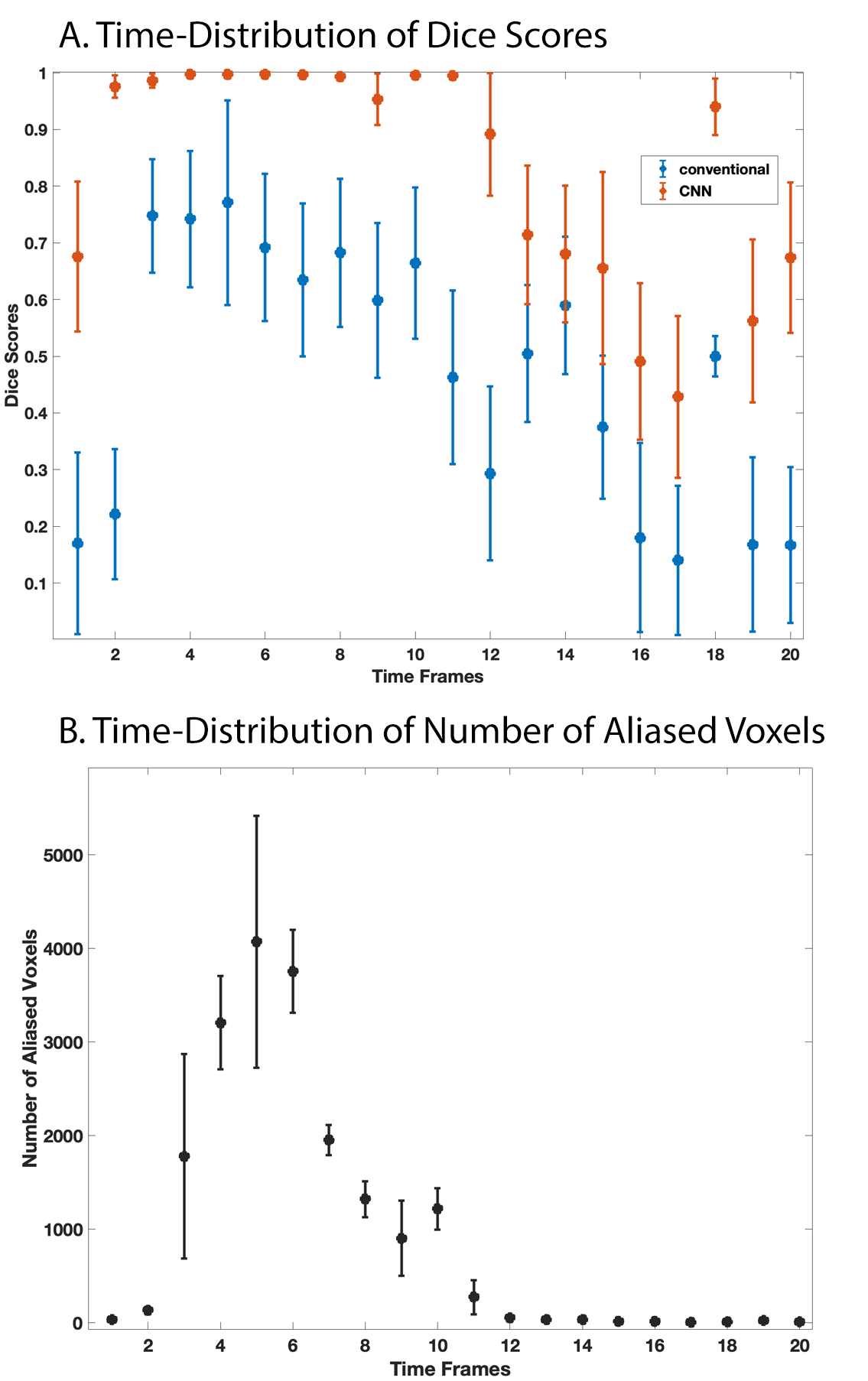


**Figure S3**: The time-distribution of Dice scores and the number of aliased voxels in the ground-truth for the 60 cm/s venc data. During systole (time-frames 3-11), the CNN performed well (median Dice score >0.9), however showed a decline in performance in diastole (time frames >11, Figure. S5, A). This is likely due to the small number of aliased voxels present in the data during diastole (Figure. S5, B) which could result in a huge impact on the Dice score as a result of missing a few voxels. The conventional algorithm showed moderate to poor performance across the cardiac cycle. The dots show the median Dice score and the bars are the interquartile range. For instances where the ground-truth was empty (no velocity aliasing), we calculated the Dice scores by adding a small constant (1e-5) at both the numerator and denominator in order to avoid dividing by zero.


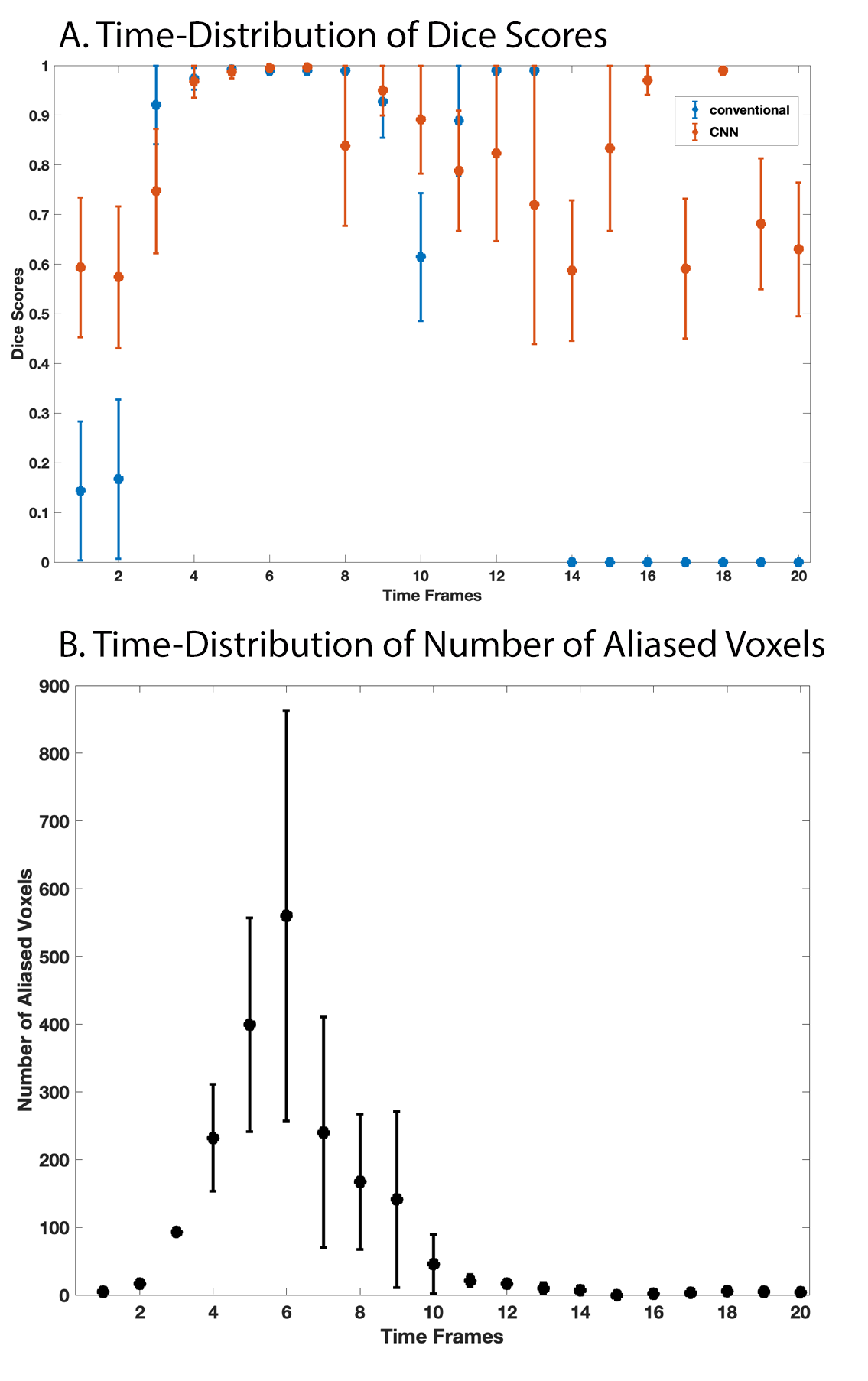


**Figure S4**: The time-distribution of Dice scores and the number of aliased voxels in the ground-truth for the 100 cm/s venc data. During systole (time-frames 3-11), the CNN performed well (median Dice score >0.9), however showed a decline in performance in diastole (time frames >11, Figure. S5, A). This is likely due to the small number of aliased voxels present in the data during diastole (Figure. S5, B) which could result in a huge impact on the Dice score as a result of missing a few voxels. The conventional algorithm, likewise, showed performance during systole but failed to detect any aliased voxels during diastole. The dots show the median Dice score and the bars are the interquartile range. For instances where the ground-truth was empty (no velocity aliasing), we calculated the Dice scores by adding a small constant (1e-5) at both the numerator and denominator in order to avoid dividing by zero.

|  | **Number of aliased voxels detected by CNN** | **Number of aliased voxels detected by Conventional**  **Algorithm** | **p-value** |
| --- | --- | --- | --- |
| **Patients with**  **Severe stenosis (N=44)** | 2141  [546-3110] | 1250  [287-2000] | <0.001 |
| **Patients with**  **Moderate-severe stenosis (N=14)** | 1175  [493-3011] | 716  [289-2005] | <0.001 |
| **Patients with**  **Moderate stenosis (N=20)** | 2010  [524-3401] | 1353  [377-2351] | <0.001 |
| **Patients with**  **Moderate-mild stenosis (N=6)** | 2128  [524-3834] | 1353  [365-2425] | <0.001 |
| **Patients with**  **Mild stenosis (N=17)** | 892  [392-3325] | 656  [234-2351] | <0.001 |
| **Patients with**  **No stenosis (N=188)** | 230  [99-718] | 102  [26-340] | <0.001 |

**Table S1**: Summary of the CNN and conventional algorithm comparison for stenosis patients with real velocity aliasing. The patients were divided into groups based on the stenotic grading. The median number of detected velocity-aliased voxels and the interquartile range are provided. There was a significant difference between the two methods across all patient groups with the CNN consistently detecting more aliased voxels than the conventional algorithm.
